# Gender and Age Influence on Emergency Department Visits for Non-Suicidal Self-Injuries in School Aged Children in Italy: An 11 Years Retrospective Cross-Sectional Study

**DOI:** 10.3389/ijph.2023.1606370

**Published:** 2023-12-18

**Authors:** Giovanni Paladini, Elena Sciurpa, Roberta Onorati, Heba Safwat Mhmoued Abdo Elhadidy, Gianmarco Giacomini, Carlo Mamo, Alberto Borraccino

**Affiliations:** ^1^ Department of Public Health Sciences and Pediatrics, University of Turin, Turin, Italy; ^2^ Regional Public Health Observatory, Epidemiology Unit, Local Health Board TO3, Grugliasco, Italy

**Keywords:** non-suicidal self-injury (NSSI), self-harm, adolescent, school-age children, emergency department

## Abstract

**Objectives:** Non-suicidal self-injury (NSSI) poses a threat in developmental ages, yet there is a scarcity of studies on NSSI trends, especially those in emergency departments (ED).

**Methods:** The aim of this cross-sectional study is to describe trends in ED visits for NSSI among young people aged between 5 and 19 years in Italy from 2011 to 2021 in Piedmont. From the ministerial ED discharge information system, all occurring NSSIs were identified by medical report and/or ICD9CM code and reported as a population rate and a visit rate on all ED requests.

**Results:** The general rate of ED visits remained stable, with around 210,000 (55% males) visits each year, along the whole period from 2011 to 2019, then halved during 2020 and 2021. The NSSI population and visits rates increased from 2013, peaking in 2019 at a rate of around 25 and 23 NSSI visits (girls) and 76 and 69 NSSI (boys) per 100,000 ED visits. In 2020 and 2021, the rate of NSSI visits increased, particularly in girls and among older adolescents.

**Conclusion:** The gradual increase of NSSI over the last decade is a rising public health issue, which deserves wider attention to ensure early detection and prevention.

## Introduction

Non suicidal self-injuries (NSSI) are recognized as a deliberate and purposeful behavior of destruction of one’s own body tissue in the absence of a lethal intent. To be an NSSI, the behavior must not be part of repetitive stereotypes and it should not be better explained by other medical conditions [1–3]. Together with suicidal ideation, which ranges from passive thoughts of death to suicide attempts [3], it is included among the self-injurious thoughts and behaviors [3, 4].

NSSI causes distress or impairment in functioning, and it is associated with both internalizing and externalizing disorders [5]. Moreover, as it is described to share the same risk factors with suicidal ideation, it may also be a predictor of suicidal behaviors [6–9].

NSSIs may be recognized as a coping strategy practiced to mitigate a negative state of mind or a specific cognitive condition (such as anxiety or tension); to overcome an interpersonal difficulty; or for inducing a perceived positive sensation [1, 10, 11]. Repetitive NSSIs appear to be more frequent than episodic NSSIs. They, indeed, represent an addictive behavior that ranges from mild forms, such as hair plucking or deliberate interference with healing processes, to more severe forms of expression, such as burning and cutting oneself [1, 11].

The NSSI phenomenon has been esteemed to have a prevalence of about 18%–22% in adolescence [12], but with a slight variability within samples and between countries [10, 12–15]. Systematic reviews further showed that the prevalence of NSSI peaks around 15/16 years of age and then declines towards late adolescence [10, 16]. Despite its occurrence, the proportion of adolescents meeting the diagnostic criteria for NSSI according to DSM-5 is much lower, between 1.5% and 6.7% [17]. According to the Global Burden of Disease (GBD, 2020) study, a collaborative project led by the Institute for Health Metrics and Evaluation (IHME), NSSIs showed an overall reduction in both prevalence and incidence of about 12.6% and 3.8% since the year 2010, however NSSIs, which amount to nearly 13.2 million cases worldwide, still represent the third leading cause of Disability-adjusted life years (DALYs) among adolescents.

Although still limited in their extension, most recent studies on the Italian population have reported conflicting results, with the prevalence of NSSIs varying widely between 12% [18, 19] to more than 40% [20] across samples. Consistently with international findings, NSSI varied by gender, with girls more commonly reported than boys, while mixed results were reported according to age difference, ethnicity, and parents’ level of education [19]. Moreover, a cross-sectional study in one of the largest children’s hospitals in Italy, on young people admitted to the Emergency Department (ED) and undergoing a child psychiatry consultation, reported an increasing trend in overall admissions for NSSI between 2011 and 2016 [21].

As a consequence of the pandemic, serious concerns have been raised about the possible impact on the mental health of adolescents, and on specific behaviors such as eating disorders and NSSIs [16, 22]. The pandemic and restrictions on social life may, indeed, have had an impact on adolescents’ health, impairing their emotional regulation and exacerbating coping strategies to stressors [22, 23]. Ultimately, they may also have been involved in the occurrence of NSSIs [23–25] especially in adolescents with pre-existing vulnerabilities [26].

In view of the aforementioned, and in relation to the limited number of large epidemiological studies on ED admissions for NSSI in young people in Italy, the aim of the study was to describe the 10 years trend in ED visits for NSSI between 2011 and 2021 in the young and adolescent population in Italy, and to discuss any potential variations between age and gender.

## Methods

### Study Population and Design

A retrospective cross-sectional observation of all ED visits among people aged 5–19 years was conducted from January 2011 to October 2021 in Piedmont, Italy. Piedmont is the second largest region in Italy, encompassing an area of approximately 25,400 square kilometres. Located in the north-western part of the country, the region hosts approximately 4.3 million inhabitants, of which nearly 15% are between 5 and 19 years of age (2021 census data). The overall population density was estimated to be about 168 inhabitants per square Kilometer.

Data on ED visits were retrieved through the National Discharge Information System (NDIS). The NDIS includes compulsory hospital and patient information—including demographics, ED visit reason, date and mode of access and of discharge, place of event and mode of occurrence, the level of severity, and the clinical diagnosis, coded according to the International Classification of Diseases 9th Revision Clinical Modification (ICD9-CM). All registered ED visits that occurred between January 2011 and October 2021 (latest information formally available for administrative use) were retrieved and included in the analysis.

The identification of NSSI events was based on the traceability of encoded information available in the NDIS, when the event was recorded by the physician through a primary or secondary diagnostic code of suspected purposely inflicted injury (codes E980-E989) or late-effects self-inflicted injury (codes E959), or specifically flagged as an intentional, non-accidental, self-inflicted injury in the dedicated field of the discharge form.

### Ethics

Study data were obtained by accessing the NDIS through the universal patient identification number (ID). The ID is a certified anonymous, non-reversible, individual code, centrally assigned before data storage. According to national data ethics regulations, access to health information is then available to accredited institutions for administrative, healthcare planning, or epidemiological purposes, and only after official institutional authorization. As such, further Ethics Committee approval is not required.

### Statistical Analyses

Descriptive analyses were performed on all ED visits in people aged 5–19 years, between the year 2011 and 2021. NSSI were grouped by gender and into three age categories, between 5 and 9, 10 to 14, and 15 to 19 years of age and then reported for each year in the period of the study as an NSSI visits rate and an NSSI population rate. The year specific NSSI visits rate and its 95% confidence intervals (CIs) were computed according to the total number of ED visits and reported as the number of cases per 100,000 visits. The year-specific NSSI population rates were computed, excluding repeated NSSI visits occurring for the same subject, and according to the age-specific population by gender, hence, they were reported as the number of cases per 100,000 inhabitants.

To account for possible differences in age distribution, and to allow year and gender comparisons, NSSI visits and population rates were derived through direct standardization using the population and distribution of ED visits in 2018, then reported as a standardized (STD) number of NSSI events every 100,000 inhabitants and STD events per 100,000 ED visits.

Any differences across age groups, gender, and year of observation were tested for significance using the McNemar test, to account for any repeated measures or clustering effect; trends over the study period were assessed using the Cochran-Armitage test and significance was set at *α* < 0.05.

Finally, to provide a synthesized measure of NSSI risk across gender, age, and years, a set of logistic regression analyses were performed on all ED visits for each year under study and overall. Results were then reported as Odds Ratios (ORs) with 95% CIs. Data management and analyses were performed using SAS version 9.4 (SAS Institute Inc., Cary, NC, United States) and STATA version 17 (^©^ Copyright StataCorp LLC 1996–2023).

## Results

**FIGURE 1:**
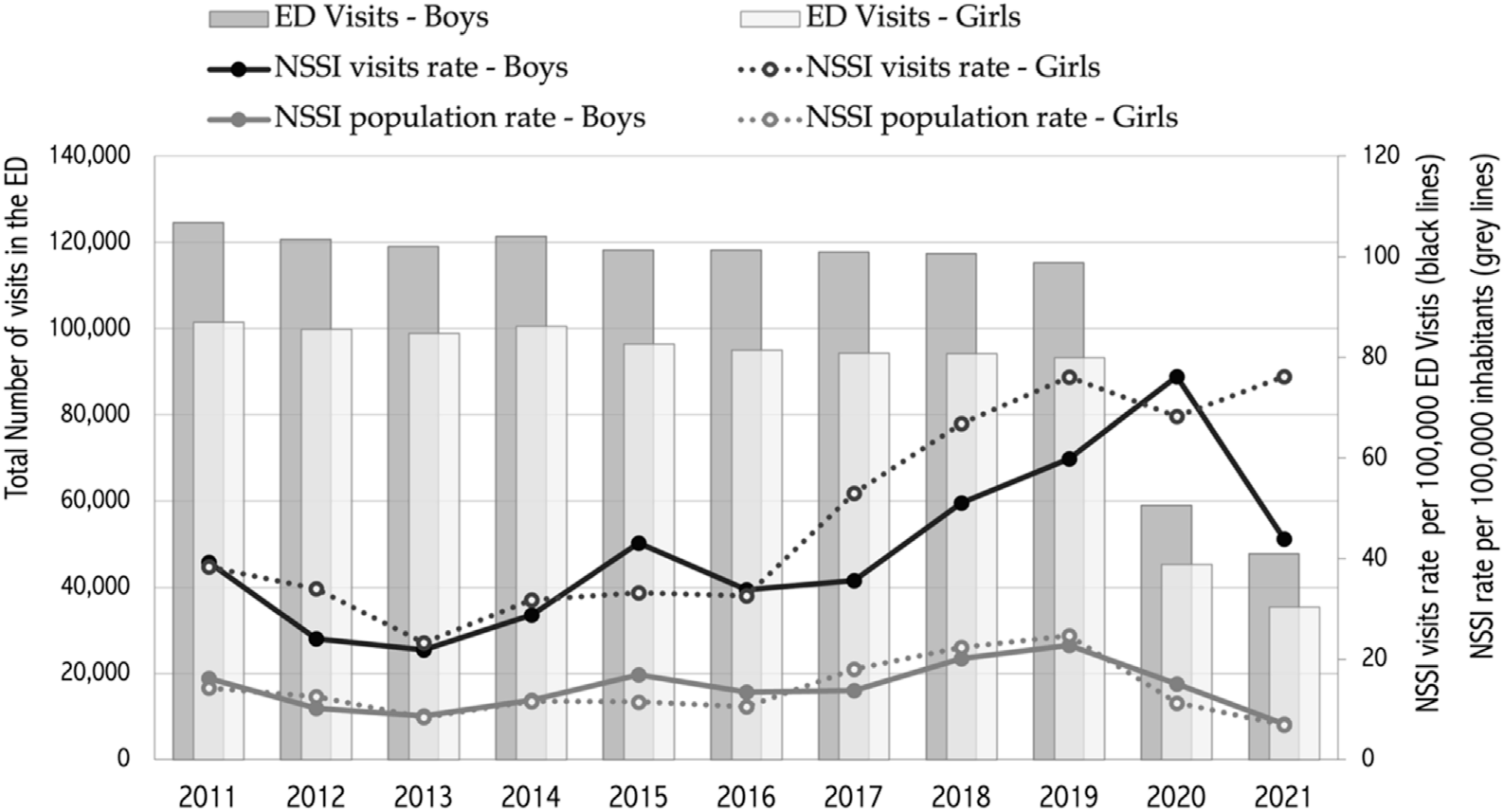
Total Number of emergency department (ED) visits, and age adjusted gender-specific (dotted line for girls) Non-Suicidal Self-Injury (NSSI) visits rate trend* per 100,000 ED visits (black lines), and NSSI rate trend per 100,000 inhabitants (grey lines) (Piedmont region, Italy, 2011–2021). NSSI, Non-Suicidal Self-Injury; ED, emergency department; * 2011–2021 Ed visits trend was significant (*p* < 0.05) over the whole period (Piedmont, Italy, 2011-2021).

NSSI admissions were assessed as a proportion per 100,000 overall ED visits among the population of those aged between 5 and 19 years old, by age groups [5–19] and gender. The rate was assessed through the last 11 years, including the main COVID-19 pandemic period, see Figure 1.

The analyses showed that ED visits were essentially stable up to 2019 with approximately 120,000 visits from boys and 100,000 from girls, as shown in Figure 1. During the pandemic and up to October 2021, the number of overall visits dropped by more than 50% (from 93,249 in 2019 to 35,451 in 2021, and from 115,306 to 47,789, respectively in girls and in boys).

The standardized NSSI population rate trend showed a slight initial decrease to the year 2013, with lowest values in the 2013 (8.7; 95% CI 5.3–12.1 and 8.4; 95% CI 4.9–11.8 for boys and girls, respectively) and an upward trend toward its peak in 2019 (22.8; 95% CI 17.3–28.3 and 24.7; 95% CI 18.9–30.6). A trend inversion was then observed in 2020 and throughout 2021, with a two- (year 2020) and three-fold (year 2021) reduction.

During the pandemic period and within the first 10 months of the year 2021, the overall number of visits to EDs decreased by more than 50% when compared to the volumes observed in the past years. This sharp reduction impacted the STD population rate, reversing the trend observed until 2019.

Comparably, the trend of the Standardized NSSI visits rate showed a slight initial decrease to the year 2013, with the lowest values in boys and girls (STD rate of 21.8; 95% CI 14.9–32.1 and 23.3; 95% CI 15.5–35.0 per 100,000 visits, respectively), then progressively, and similarly in both genders, it rose, peaking in the year 2020 in boys (STD ratio of 76.2; 95% CI 56.9–102.0) and in the year 2021 in girls (STD ratio of 76.2; 95% CI 52.2–111.0). For both genders an upward significant (*p* < 0.05) trend over the whole period was observed for NSSI ED visits (Figure 1).

As reported in Tables 1, 2, the rate of NSSI visits by age group revealed different patterns in boys and girls and among age groups. Adolescents between 15 and 19 years of age reported the highest NSSI rate in both genders in each year. Girls showed an initial drop by about two-thirds between 2011 and 2013 (from a rate of 61.6, 95% CI 40.9–92.7 to a rate of 19.5 per 100,000 ED visits, 95% CI 9.3–41.0), then increased by more than five times in 2020 to a rate of 112.9 (95% CI 72.0–177.0), before decreasing to a rate of 104.6 (95% CI 62.0–176.5) in 2021.

**TABLE 1:** Number of overall emergency department (ED) visits and of non-suicidal self-injuries (NSSI), and age category-specific NSSI rate, per 100,000 ED visits, with 95% Confidence Interval (95% CI) per year in Girls (Piedmont, Italy, 2011–2021).

Girls	5–9 years of age			10–14 years of age			15–19 years of age		
Year	Visits	NSSI	Rate (95% CI)	Visits	NSSI	Rate (95% CI)	Visits	NSSI	Rate (95% CI)
2011	33,158	8	24.13 (12.1–48.2)	30,988	8	25.82 (12.9–51.6)	37,334	23	61.61 (40.9–92.7)
2012	33,055	8	24.20 (12.1–48.4)	30,855	5	16.20 (6.8–38.9)	35,963	21	58.39 (38.1–89.5)
2013	32,085	9	28.05 (14.6–53.9)	30,976	7	22.60 (10.8–47.4)	35,803	7	19.55 (9.3–41.0)
2014	32,814	9	27.43 (14.3–52.7)	31,910	8	25.07 (12.5–50.1)	35,850	15	41.84 (25.2–69.4)
2015	32,162	11	34.20 (18.9–61.8)	30,192	10	33.12 (17.8–61.6)	34,034	11	32.32 (17.9–58.4)
2016	32,797	5	15.25 (6.4–36.6)	29,305	11	37.54 (20.8–67.8)	32,947	15	45.53 (27.5–75.5)
2017	31,591	11	34.82 (19.3–62.9)	29,797	20	67.12 (43.3–104.0)	32,971	19	57.63 (36.8–90.3)
2018	31,165	17	51.34 (33.9–87.7)	30,256	20	66.10 (42.7–102.4)	32,826	26	79.21 (53.9–116.3)
2019	30,395	19	62.51 (39.9–98.0)	30,404	19	62.49 (39.9–98.0)	32,450	33	101.69 (72.3–143.0)
2020	14,072	3	21.32 (6.9–66.1)	14,524	9	61.97 (32.3–119.1)	16,825	19	112.93 (72.0–177.0)
2021	10,136	2	19.73 (4.9–78.9)	11,928	11	92.22 (51.1–166.4)	13,387	14	104.58 (62.0–176.5)

**TABLE 2:** Number of overall emergency department (ED) visits and of non-suicidal self-injuries (NSSI), and age category-specific NSSI rate, per 100,000 ED visits, with 95% Confidence Interval (95% CI) per year in Boys (Piedmont, Italy, 2011–2021).

Boys	5–9 years of age			10–14 years of age			15–19 years of age		
Year	Visits	NSSI	Rate (95% CI)	Visits	NSSI	Rate (95% CI)	Visits	NSSI	Rate (95% CI)
2011	42,635	16	37.53 (23.0–61.3)	42,488	14	32.95 (19.5–55.6)	39,484	19	48.12 (30.7–75.4)
2012	42,263	8	18.93 (9.5–37.9)	41,637	5	12.01 (5.0–28.9)	36,822	16	43.45 (26.6–70.9)
2013	41,381	6	14.5 (6.5–32.3)	41,798	9	21.53 (11.2–41.4)	35,883	11	30.66 (17.0–55.4)
2014	42,344	12	28.34 (16.1–49.9)	42,780	6	14.03 (6.3–31.2)	36,311	17	46.82 (29.1–75.3)
2015	42,060	17	40.42 (25.1–65.0)	40,711	13	31.93 (18.5–55.0)	35,436	21	59.26 (38.6–90.9)
2016	41,963	11	26.21 (14.5–47.3)	40,463	13	32.13 (18.7–55.3)	35,807	16	44.68 (27.4–72.9)
2017	40,217	12	29.84 (17.0–52.5)	41,401	14	33.82 (20.0–57.1)	36,132	16	44.28 (27.1–72.3)
2018	40,334	23	57.02 (37.9–85.8)	41,343	17	41.12 (25.6–66.1)	35,681	20	56.05 (36.2–86.9)
2019	38,231	16	41.85 (25.6–68.3)	41,719	23	55.13 (36.6–83.0)	35,356	30	84.85 (59.3–121.3)
2020	18,511	9	48.62 (25.3–93.4)	20,755	18	86.73 (54.7–137.6)	19,798	18	90.92 (57.3–144.3)
2021	13,312	7	52.58 (25.1–110.3)	17,707	9	50.83 (26.5–97.7)	16,770	5	29.82 (12.4–71.6)

Similarly, the boys reported an initial drop in the NSSI rate from 48.1 (95% CI 30.7–75.4) to 30.7 (95% CI 17.0–55.4) NSSI between 2011 and 2013, then increased to 90.9 (95% CI 57.3–144.3) in 2020, before dropping to the lowest value of 29.8 (95% CI 12.4–71.6) NSSI per 100,000 ED visits.

In the 10 to 14 age group, the NSSI rate showed—in both genders—a continuous upward trend from 2012 to 2020 (from a rate of 12.0 to 86.7 NSSI per 100,000 visits; 95% CI 5.0–28.9 and 54.7–137.6, respectively, in boys, and from a rate of 16.2 to 22.2 in 2021 in girls; 95% IC 6.8–38.9 and 51.1–166.4).

The youngest age group reported the lowest rates along the whole period under study, peaking in 2019 in girls (NSSI rate of 62.5; 95% CI 39.9–98.0), then followed by a drop in 2020 which remained nearly stable in 2021, and peaking in 2018 in boys (57.0; 95% CI 37.9–85.8), followed by a slight decrease in 2019, before increasing again to about 52.6 (95% CI 25.1–110.3) in 2021.

Table 3 finally reported the yearly and overall Odds (and 95% Confidence Interval) of NSSI visits in girls compared to boys, and in the age groups of 10–14 and 15–19 years of age compared to the youngest age group.

**TABLE 3:** Odds ratios (OR) and 95% Confidence interval (95% CI) of non-suicidal self-injuries (NSSI) by gender and age category per year and overall^a^ (Piedmont, Italy, 2011–2021).

Year	Girls	10–14 years of age	15–19 years of age
	OR (95% CI)	OR (95% CI)	OR (95% CI)
2011	0.95 (0.6–1.4)	0.94 (0.5–1.7)	**1.73 (1.1–2.9)**
2012	1.32 (0.8–2.2)	0.65 (0.3–1.4)	**2.36 (1.3–4.2)**
2013	1.05 (0.6–1.9)	1.08 (0.5–2.2)	1.23 (0.6–2.4)
2014	1.05 (0.7–1.7)	0.67 (0.3–1.3)	1.58 (0.9–2.7)
2015	0.76 (0.5–1.2)	0.86 (0.5–1.5)	1.24 (0.7–2.1)
2016	0.95 (0.6–1.5)	1.61 (0.9–3.0)	**2.11 (1.2–3.9)**
2017	1.47 (1.0–2.2)	1.50 (0.9–2.6)	1.56 (0.9–2.6)
2018	1.29 (0.9–1.8)	0.93 (0.6–1.4)	1.19 (0.8–1.8)
2019	1.20 (0.9–1.7)	1.15 (0.7–1.8)	**1.81 (1.2–2.7)**
2020	0.88 (0.6–1.4)	**2.07 (1.1–4.1)**	**2.75 (1.4–5.3)**
2021	1.74 (1.0–3.1)	1.79 (0.8–3.9)	1.63 (0.7–3.6)
Overall	1.13 (1.0–1.3)	1.12 (0.9–1.3)	**1.65 (1.4–1.9)**

^a^ Reference categories for gender: boys, and “5–9 years of age” for age category. Yearly OR are adjusted for age category and gender, and overall OR are adjusted age category, gender and year.

Statistically significant values are in bold.

Regardless of age, the analyses showed a slight, but non-significant, excess risk of NSSI in girls with an overall OR of about 1.13 (95% CI 1.0–1.3). However, significant differences were reported within the age groups and, in particular, in the 15–19 years of age group, with higher Odds of NSSI along the 11 years study period, varying between 1.2 and 2.8 and an overall significant risk of 1.65 (95% CI 1.4–1.9) for the whole period. During the 2020, adolescents between 10 to 14 and 15 to 19 years of age showed a significant OR of visiting ED for NSSIs of respectively 2.07 (95% CI 1.1–4.1) and 2.75 (95% CI 1.4–5.3), with a non-significant lower risk for girls (OR 0.88; 95% CI 0.6–1.4).

## Discussion

This study assessed the trend of ED visits over the past 11 years with the aim of discussing the occurrence and trend of visits for NSSI, highlighting the impact that the COVID-19 period may have had on it. Our results showed that, after a long period of relative stability in overall ED visits, a sharp decline of more than half of the expected numbers was observed by 2020 onwards. This finding is consistent with previous studies worldwide and in Italy, as both routine and emergency care were greatly reduced due to the pandemic [22, 27].

Along with the overall reduction in ED visits, the pandemic had also impacted on the type of requests, by reducing those that were deferrable or perceived as less urgent [22]. As a result, specific ED requests, which represented a relative minority in the overall distribution of accesses, appeared to be consistently more frequent during the decline of overall visits in 2020 and 2021. The remarkable difference observed in NSSI population rates and in the rate of NSSI visits, represents the complexity of the situation quite well. The impact of the pandemic on overall ED visits has produced a spurious decrease in NSSI population rates, while over the same period, health professionals were overwhelmed by the relative unexpected increase in NSSI occurrence.

Consistently with other recent studies, indeed, our results confirmed the relative increase in NSSIs during the pandemic [17], but in contrast to the GBD study, which showed a slight decrease in self-harm between 2010 and 2019 (GBD, 2020), we also observed that the rate of NSSI visits was already increasing since 2013, both at the population and at the ED visits level, and especially in the youth and adolescent age group.

Focusing on the period of the COVID-19 pandemic, a recent systematic review reported that despite the decrease in the absolute frequency of events, ED visits for NSSI have proportionally increased, along with the severity of the treated events, suggesting that the pandemic may have acted as a trigger on youth mental health. The same work, indeed, reported an increased use of health services following acts of self-harm, especially among adolescents, and particularly among girls [25]. Similarly, other recent studies observed that the pandemic had severely impacted on those adolescents who, for individual or social reasons, were identifiable as more vulnerable. For these adolescents the increased difficulty in coping with emerging stressors, such as loneliness, reduced social support, limited opportunities to engage in social activities, or simply pursue personal interests, may have contributed to the higher likelihood of engaging in NSSI [24, 26, 28–30].

As regards observed gender differences, our results revealed a slight non-significant difference in NSSI along the period under study and overall. However, important differences emerged in gender patterns and among age categories. The NSSI visit rate peaked in boys during the pandemic years and then declined, while it continued to increase in girls, highlighting a different attitude to this issue which, despite its statistical significance, deserves more attention. This difference, in fact, could be explained by the different timings of developmental stages between boys and girls in adolescence, as girls tends to reveal effects of stressors earlier in life than coetaneous boys [31]. In addition, boys are more likely to mask possible signs of self-inflicted injuries (e.g., by attributing them to the consequences of sporting activities) and less likely to seek help than girls of the same age [12], which, due to the interruption of sporting activities and the greater presence of relatives or carers at home, could partly explain the differences observed during the pandemic.

With regard to age group, the reported difference in age-specific NSSI visit rates observed among the older age groups—well summarized in the significant risk levels—seems to be in line with available scientific findings that also reported a higher occurrence of NSSI in adolescents attending high-school than in younger students [15, 32]. This difference could be associated with the different developmental stages in growth, and in particular of the prefrontal cortex during adolescence, which, still in its plastic phase, provides altered emotional control of stressors. According to Wilkinson, this seems to be the pattern in adolescence and early adulthood, where a higher rate of NSSIs is consistently observed compared to younger and to older ages. Moreover, as NSSIs are often part of a broader maladaptive coping strategy, this result is consistent with a general increase in psychological distress observed in the same age groups [8]. According to other studies, indeed, the psychological characteristics more frequently associated with NSSI behaviors are poorer emotional regulation, poorer academic performance, interpersonal difficulties (e.g., high sensitivity to rejection), and a negative attributional style [33, 34].

Given this steady 10 years increase in self-harm and the fact that NSSI episodes occurring in EDs represent only the emerging and visible part of all instances of self-harm, it becomes increasingly urgent to focus public health efforts on improving both early detection and the ability to successfully prevent them. The heterogeneity of the results available in the literature—in which there are different approaches to the estimation of NSSI and consequently the overall concerns on the phenomenon—reveals that more attention needs to be paid to these behaviors.

According to WHO data, NSSIs are themselves a risk factor for suicide in young people, which is reported to be a leading cause of death in young people [35]. As such, NSSIs could have a significant impact on adolescents’ health development. As a young person’s identity develops during adolescence, it establishes the precondition for mental wellbeing [36]. In this context, conducts such as NSSIs that may arise early in growth and worsen over time need to be addressed early [37]. It is therefore necessary to improve and standardize the use of available information sources in order to rapidly identify young people at risk of NSSI and offer gender- and age-appropriate treatment pathways. At the same time, it is necessary to improve the ability to use these sources of information to increase knowledge of the possible factors surrounding NSSI, on an individual, environmental, or social level.

### Limitations

Although our study was modestly constructed using a descriptive approach and made use of currently available sources of information, which is an overall strength, it is also necessary to consider its results in light of its limitations.

As the study is based on official ministerial administrative data, which are intrinsically solid and exhaustive, they can suffer in accuracy as data entry is operator dependent. In addition, the criteria used to define whether an observed event was intentional or not may vary according to several factors such as individual sensitivity or attention, interviewing ability, time available for assessment, and so on. A further limitation is related to 2021 data availability, which, due to legal procedures, was limited to the month of October. Therefore, the occurrence of NSSI identified could potentially be underestimated.

Finally, for the purposes of the study, our analysis included all ED visits, regardless of whether they were first-time or repeated NSSI visits (which in any case accounted for less than 3% of all visits) as they represent the hospital burden associated with the phenomenon under study.

### Conclusion

The increase in NSSI episodes over time, in contrast to the gradual decrease in ED visits, highlights the importance of better analyzing these trends in order to understand the hidden patterns of the NSSI phenomenon. Over the past decade, both the population rate and the rate of ED visits for NSSI have increased, well before the COVID-19 pandemic, which had a negative impact on the population rate while, simultaneously, exacerbating the phenomenon. NSSIs in the young population are a non-deferrable emergency, representing a serious a public health concern. It is of great importance to promote the improvement of both early treatment and prevention in primary healthcare services, in order to promptly detect and avoid worse outcomes. To date, NSSI may be only one of the phenomena reflecting the deterioration of mental health in children and adolescents. These trends should be studied through longitudinal approaches and constantly monitored in order to improve adolescents and population health.

## Data Availability

The datasets generated and/or analyzed during the current study are not publicly available. Data are accessible upon specific request to the study data manager, Roberta Onorati, roberta.onorati@epi.piemonte.it.

## References

[1] American Psychiatric Assosiation. Diagnostic and Statistical Manual of Mental Disorders: DSM-5. Association AP, Force APAssociationD 5 TM. Arlington, VA: American Psychiatric Association (2013).

[2] Klonsky ED. The Functions of Deliberate Self-Injury: A Review of the Evidence. Clin Psychol Rev (2007) 27(2):226–39. 10.1016/j.cpr.2006.08.002 17014942

[3] Bettis AH Benningfield MM Dao A Dickey L Pegg S Venanzi L Self-Injurious Thoughts and Behaviors and Alterations in Positive Valence Systems: A Systematic Review of the Literature. J Psychiatr Res (2022) 156:579–93. 10.1016/j.jpsychires.2022.10.033 36370537 PMC9742322

[4] Cha CB Wilson KM Tezanos KM DiVasto KA Tolchin GK. Cognition and Self-Injurious Thoughts and Behaviors: A Systematic Review of Longitudinal Studies. Clin Psychol Rev (2019) 69:97–111. 10.1016/j.cpr.2018.07.002 30166197

[5] Meszaros G Horvath LO Balazs J. Self-Injury and Externalizing Pathology: A Systematic Literature Review. BMC Psychiatry (2017) 17(1):160. 10.1186/s12888-017-1326-y 28468644 PMC5415783

[6] Asarnow JR Porta G Spirito A Emslie G Clarke G Wagner KD Suicide Attempts and Nonsuicidal Self-Injury in the Treatment of Resistant Depression in Adolescents: Findings From the TORDIA Study. J Am Acad Child Adolesc Psychiatry (2011) 50(8):772–81. 10.1016/j.jaac.2011.04.003 21784297 PMC3143365

[7] Tang J Yu Y Wu Y Du Y Ma Y Zhu H Association Between Non-Suicidal Self-Injuries and Suicide Attempts in Chinese Adolescents and College Students: A Cross-Section Study. PLoS One (2011) 6(4):e17977. 10.1371/journal.pone.0017977 21494656 PMC3072963

[8] Wilkinson P Kelvin R Roberts C Dubicka B Goodyer I. Clinical and Psychosocial Predictors of Suicide Attempts and Nonsuicidal Self-Injury in the Adolescent Depression Antidepressants and Psychotherapy Trial (ADAPT). Am J Psychiatry (2011) 168:495–501. 10.1176/appi.ajp.2010.10050718 21285141

[9] Mars B Heron J Klonsky ED Moran P O’Connor RC Tilling K Predictors of Future Suicide Attempt Among Adolescents With Suicidal Thoughts or Non-Suicidal Self-Harm: A Population-Based Birth Cohort Study. Lancet Psychiatry (2019) 6(4):327–37. 10.1016/S2215-0366(19)30030-6 30879972 PMC6494973

[10] Cipriano A Cella S Cotrufo P. Nonsuicidal Self-Injury: A Systematic Review. Front Psychol (2017) 8:1946. 10.3389/fpsyg.2017.01946 29167651 PMC5682335

[11] Halicka J Kiejna A. Non-Suicidal Self-Injury (NSSI) and Suicidal: Criteria Differentiation. Adv Clin Exp Med (2018) 27(2):257–61. 10.17219/acem/66353 29521070

[12] Esposito C Dragone M Affuso G Amodeo AL Bacchini D. Prevalence of Engagement and Frequency of Non-Suicidal Self-Injury Behaviors in Adolescence: An Investigation of the Longitudinal Course and the Role of Temperamental Effortful Control. Eur Child Adolesc Psychiatry (2022) 32:2399–414. 10.1007/s00787-022-02083-7 36123505 PMC10682258

[13] Xiao Q Song X Huang L Hou D Huang X. Global Prevalence and Characteristics of Non-Suicidal Self-Injury Between 2010 and 2021 Among a Non-Clinical Sample of Adolescents: A Meta-Analysis. Front Psychiatry (2022) 13:912441. 10.3389/fpsyt.2022.912441 36032224 PMC9399519

[14] Swannell SV Martin GE Page A Hasking P St John NJ. Prevalence of Nonsuicidal Self-Injury in Nonclinical Samples: Systematic Review, Meta-Analysis and Meta-Regression. Suicide Life Threat Behav (2014) 44(3):273–303. 10.1111/sltb.12070 24422986

[15] Muehlenkamp JJ Gutierrez PM. An Investigation of Differences Between Self-Injurious Behavior and Suicide Attempts in a Sample of Adolescents. Suicide Life Threat Behav (2004) 34(1):12–23. 10.1521/suli.34.1.12.27769 15106884

[16] Plener PL. COVID-19 and Nonsuicidal Self-Injury: The Pandemic’s Influence on an Adolescent Epidemic. Am J Public Health (2021) 111:195–6. 10.2105/AJPH.2020.306037 33439716 PMC7811075

[17] Gillies D Christou MA Dixon AC Featherston OJ Rapti I Garcia-Anguita A Prevalence and Characteristics of Self-Harm in Adolescents: Meta-Analyses of Community-Based Studies 1990-2015. J Am Acad Child Adolesc Psychiatry (2018) 57(10):733–41. 10.1016/j.jaac.2018.06.018 30274648

[18] Gatta M Rago A Dal Santo F Spoto A Battistella P. Non-Suicidal Self-Injury Among Northern Italian High School Students: Emotional, Interpersonal and Psychopathological Correlates. Original article J Psychopathology (2016) 22:185–90.

[19] Giletta M Scholte RHJ Engels RCME Ciairano S Prinstein MJ. Adolescent Non-Suicidal Self-Injury: A Cross-National Study of Community Samples From Italy, the Netherlands and the United States. Psychiatry Res (2012) 197(1–2):66–72. 10.1016/j.psychres.2012.02.009 22436348 PMC3666103

[20] Cerutti R Manca M Presaghi F Gratz KL. Prevalence and Clinical Correlates of Deliberate Self-Harm Among a Community Sample of Italian Adolescents. J Adolesc (2011) 34(2):337–47. 10.1016/j.adolescence.2010.04.004 20471075

[21] Castaldo L Serra G Piga S Reale A Vicari S. Suicidal Behaviour and Non-Suicidal Self-Injury in Children and Adolescents Seen at an Italian Paediatric Emergency Department. Ann Ist Super Sanita (2020) 56:303–14. 10.4415/ANN_20_03_08 32959796

[22] Giacomini G Elhadidy HSMA Paladini G Onorati R Sciurpa E Gianino MM Eating Disorders in Hospitalized School-Aged Children and Adolescents During the COVID-19 Pandemic: A Cross-Sectional Study of Discharge Records in Developmental Ages in Italy. Int J Environ Res Public Health (2022) 19:12988. 10.3390/ijerph192012988 36293569 PMC9602016

[23] Robillard CL Turner BJ Ames ME Craig SG. Deliberate Self-Harm in Adolescents During COVID-19: The Roles of Pandemic-Related Stress, Emotion Regulation Difficulties, and Social Distancing. Psychiatry Res (2021) 304:114152. 10.1016/j.psychres.2021.114152 34371298 PMC8424292

[24] Du N Ouyang Y Xiao Y Li Y. Psychosocial Factors Associated With Increased Adolescent Non-Sicidal Self-Injury During the COVID-19 Pandemic. Front Psychiatry (2021) 12:743526. 10.3389/fpsyt.2021.743526 34955911 PMC8703160

[25] Steeg S John A Gunnell DJ Kapur N Dekel D Schmidt L The Impact of the COVID-19 Pandemic on Presentations to Health Services Following Self-Harm: Systematic Review. Br J Psychiatry (2022) 221:603–12. 10.1192/bjp.2022.79 35816104

[26] De Luca L Giletta M Nocentini A Menesini E. Non-Suicidal Self-Injury in Adolescence: The Role of Pre-Existing Vulnerabilities and COVID-19-Related Stress. J Youth Adolesc (2022) 51(12):2383–95. 10.1007/s10964-022-01669-3 35986870 PMC9392436

[27] Cassell K Zipfel CM Bansal S Weinberger DM. Trends in Non-COVID-19 Hospitalizations Prior to and During the COVID-19 Pandemic Period, United States, 2017-2021. Nat Commun (2022) 13:5930. 10.1038/s41467-022-33686-y 36209210 PMC9546751

[28] Carosella KA Wiglesworth A Silamongkol T Tavares N Falke CA Fiecas MB Non-Suicidal Self-Injury in the Context of COVID-19: The Importance of Psychosocial Factors for Female Adolescents. J Affect Disord Rep (2021) 4:100137. 10.1016/j.jadr.2021.100137

[29] Gruber J Prinstein MJ Clark LA Rottenberg J Abramowitz JS Albano AM Mental Health and Clinical Psychological Science in the Time of COVID-19: Challenges, Opportunities, and a Call to Action. Am Psychol (2021) 76(3):409–26. 10.1037/amp0000707 32772538 PMC7873160

[30] Zetterqvist M Landberg A Jonsson LS Svedin CG. The Psychosocial Consequences of COVID-19 in Adolescents With Nonsuicidal Self-Injury. Child Adolesc Psychiatry Ment Health (2023) 17(1):33. 10.1186/s13034-023-00566-2 36871031 PMC9985473

[31] Riva D. Sex and Gender Difference in Cognitive and Behavioral Studies in Developmental Age: An Introduction. J Neurosci Res (2023) 101:543–52. 10.1002/jnr.24970 34687075

[32] Whitlock J Eckenrode J Silverman D. Self-Injurious Behaviors in a College Population. Pediatrics (2006) 117(6):1939–48. 10.1542/peds.2005-2543 16740834

[33] Barrocas AL Giletta M Hankin BL Prinstein MJ Abela JRZ. Nonsuicidal Self-Injury in Adolescence: Longitudinal Course, Trajectories, and Intrapersonal Predictors. J Abnorm Child Psychol (2015) 43(2):369–80. 10.1007/s10802-014-9895-4 24965674

[34] Sintes A Fernández M Puntí J Soler J Santamarina P Soto À Review and Update on Non-Suicidal Self-Injury: Who, How and Why. Actas Esp Psiquiatr (2018) 46(4):146–55. 30079928

[35] World Health Organization Regional Office for the Eastern Mediterranean. Suicide and Self-Harm. Mediterranean: World Health Organization (2019). Available From: https://apps.who.int/iris/handle/10665/333478 (Accessed June 6, 2023).

[36] Van Dijk MPA Hale WW Hawk ST Meeus W Branje S. Personality Development From Age 12 to 25 and Its Links With Life Transitions. Eur J Pers (2020) 34:322–44. 10.1002/per.2251

[37] Grandclerc S De Labrouhe D Spodenkiewicz M Lachal J Moro MR. Relations Between Nonsuicidal Self-Injury and Suicidal Behavior in Adolescence: A Systematic Review. PLoS One (2016) 11:e0153760. 10.1371/journal.pone.0153760 27089157 PMC4835048

